# Uso de Terapia Anticoagulante em Obesos: Qual a Evidência da Dose Ideal?

**DOI:** 10.36660/abc.20240496

**Published:** 2024-09-17

**Authors:** Marcia M. Noya-Rabelo, Eduardo Novaes, Renata Moll-Bernardes, Olga Souza

**Affiliations:** 1 Hospital Aliança Salvador BA Brasil Hospital Aliança, Salvador, BA – Brasil; 2 Instituto D’Or de Ensino e Pesquisa Salvador BA Brasil Instituto D’Or de Ensino e Pesquisa (IDOR), Salvador, BA – Brasil; 3 Escola Bahiana de Medicina e Saúde Pública Salvador BA Brasil Escola Bahiana de Medicina e Saúde Pública (EBMSP), Salvador-BA – Brasil

**Keywords:** Anticoagulantes, Enoxaparina, Fondaparinux, Obesidade, Síndrome Coronariana Aguda

Dados da Organização Mundial de Saúde apontam para mais de 1 bilhão de pessoas no mundo, uma a cada oito, com obesidade.^1^ No Brasil, segundo pesquisa da Vigilância de Fatores de Risco de Doenças Crônicas por Inquérito Telefônico (VIGITEC), uma a cada quatro pessoas da população adulta é obesa.^2^ Obesidade é definida por índice de massa corporal (IMC) maior ou igual a 30 kg/m^2^, enquanto obesidade mórbida é considerada para aqueles com IMC acima de 40 kg/m^2^. Dados epidemiológicos também pontuam alta prevalência de doença coronariana na população brasileira estimada em 5 a 8%.^3^

Embora as taxas de obesidade atinjam proporções epidêmicas em todo o mundo, a literatura médica carece de dados precisos sobre alterações na farmacocinética e farmacodinâmica das diversas medicações em pacientes com obesidade. As possíveis diferenças fisiológicas entre obesos e não obesos podem resultar em diferenças na distribuição e eliminação de fármacos, fatores importantes a serem considerados na determinação da posologia adequada para o tratamento farmacológico. A dose ideal da maioria dos anticoagulantes para os obesos não foi estabelecida, incluindo heparina de baixo peso molecular e pentassacarídeos. Dessa forma, o manejo terapêutico dos pacientes com síndrome coronariana aguda (SCA) e obesidade é desafiador e, consequentemente, pode influenciar a eficácia e a segurança dos anticoagulantes.

A dose ideal de enoxaparina é calculada com base no peso do paciente; entretanto, como não há distribuição da droga na gordura, existe a possibilidade de exposição excessiva ao efeito anticoagulante em obesos, o que contribui para a preocupação de concentrações supra terapêuticas quando utilizamos dose baseada no peso corporal.^4^ Já o fondaparinux é um pentassacarídeo sintético que atua como um inibidor seletivo do fator Xa tendo como vantagem a dose fixa, independente do peso corpóreo. Até o momento, poucos estudos foram realizados em pacientes obesos com a finalidade de testar o ajuste posológico, nenhum deles na população com SCA.

Nesta edição dos Arquivos Brasileiros de Cardiologia, em estudo de coorte retrospectivo, Darzé et al., compararam uso de enoxaparina e fondaparinux em 367 pacientes obesos internados com SCA, no período de 2010 a 2020. Foi definido como desfecho primário a combinação de mortalidade por todas as causas, reinfarto, acidente vascular cerebral e sangramento maior durante a hospitalização. Os autores não encontraram diferença no desfecho composto destacando a possibilidade de uso de fondaparinux ser tão efetiva quanto enoxaparina sem a necessidade de ajuste de dose.^5^

Alguns especialistas recomendam medir o fator anti-Xa em obesos, no entanto, o nível mínimo de atividade necessário para que o tratamento seja eficaz não foi bem definido. Os níveis alvo máximos de anti-Xa para dosagem duas vezes ao dia são normalmente 0,5 ou 0,6-1,0 UI/mL e podem ser utilizados como marcador substituto de eficácia e segurança terapêutica.^4^ O primeiro estudo a definir uma dose ideal foi uma série de casos descritos por Deal et al.^6^ que determinaram que a dose inicial para atingir o nível ideal de anti-Xa seria de 0,74 mg/kg em pacientes com IMC médio de 49,5 kg/m^2^. Estudos subsequentes definiram doses ótimas variando de 0,70 a 0,81 mg/kg por dose.^7^ Essa variabilidade provavelmente se deve a vários fatores: primeiro, parece que mesmo dentro de uma população de pacientes obesos, aqueles com IMC mais elevados necessitam de menor dose total para atingir níveis ideais de anti-Xa, particularmente em pacientes com IMC superior a 50 kg/m^2^; em segundo lugar, a variabilidade pode ser resultante do momento da mensuração do nível de anti-Xa.^8^

Para responder as questões anteriores Chilbert et al.,^9^ em revisão sistemática, avaliaram os níveis de anti-Xa em pacientes obesos recebendo terapia com enoxaparina na dose padrão (≥ 0,95 mg/kg) versus dosagens reduzidas que foram agrupadas em <0,75 mg/kg (muito baixa) e 0,75–0,85 mg/kg (baixa). Os pacientes que receberam 0,75–0,85 mg/kg apresentaram mais resultados na faixa terapêutica sem aumento aparente no risco trombótico, enquanto os pacientes que receberam ≥0,95 mg/kg tiveram aumento de eventos hemorrágico. Se for indicada a monitorização do anti-fator Xa, o nível de atividade deve ser determinado três a cinco horas após a dose e apenas depois do paciente ter recebido pelo menos duas doses. Recomenda-se monitorização clínica rigorosa de sinais e sintomas de complicações, como hemorragia ou trombose, particularmente naqueles com peso >150 kg ou IMC >40 kg/m^2^.^10,11^

Frente à escassez de dados, mais estudos são necessários, como o realizado por Darzé et al.,^5^ para fornecer diretrizes sobre a melhor abordagem para o uso adequado de anticoagulantes em pacientes obesos com SCA, garantindo assim a otimização dos resultados clínicos e a segurança dos pacientes. A enoxaparina oferece a vantagem de dosagem ajustada ao peso, o que pode ser crucial em alguns casos, mas requer uma vigilância mais rigorosa para evitar complicações hemorrágicas. O fondaparinux, com sua dosagem fixa e menor risco de sangramento, apresenta uma opção atraente, especialmente para pacientes com maior risco de eventos hemorrágicos. Sendo assim, a decisão terapêutica para o uso de anticoagulante em obesos, deve ser respaldada em uma abordagem holística, considerando as particularidades com atenção especial à função renal, histórico de sangramento e risco trombótico, visando maximizar os benefícios terapêuticos e minimizar os riscos.
