# Supplementary material for: Uso de Terapia Anticoagulante em Obesos: Qual a Evidência da Dose Ideal?
Source: Arq Bras Cardiol. 2024 Sep 17;121(8):e20240496. [Article in Portuguese] doi: 10.36660/abc.20240496 (PMC11495805; doi:10.36660/abc.20240496)
Supplement: Translation

# Translation: Use of Anticoagulant Therapy in Obese People: What is the Evidence for the Ideal Dose?

**DOI:** 10.36660/abc.20240496i

**Authors:** Marcia M. Noya-Rabelo, Eduardo Novaes, Renata Moll-Bernardes, Olga Souza

**Affiliations:** 1 Salvador BA Brazil Hospital Aliança, Salvador, BA – Brazil; 2 Salvador BA Brazil Instituto D’Or de Ensino e Pesquisa (IDOR), Salvador, BA – Brazil; 3 Salvador BA Brazil Escola Bahiana de Medicina e Saúde Pública (EBMSP), Salvador-BA – Brazil

**Keywords:** Anticoagulants, Enoxaparin, Fondaparinux, Obesity, Acute Coronary Syndrome

Data from the World Health Organization indicate that more than 1 billion people in the world, one in eight, are obese.^1^ In Brazil, according to research by the Chronic Disease Risk Factor Surveillance by Telephone Survey (VIGITEC), one in every four people in the adult population is obese.^2^ Obesity is defined by a body mass index (BMI) greater than or equal to 30 kg/m^2^, while morbid obesity is considered for those with a BMI above 40 kg/m^2^. Epidemiological data also points to a high prevalence of coronary disease in the Brazilian population, estimated at 5 to 8%.^3^

Although obesity rates have reached epidemic proportions worldwide, the medical literature lacks precise data on changes in the pharmacokinetics and pharmacodynamics of different medications in patients with obesity. Possible physiological differences between obese and non-obese individuals may result in differences in the distribution and elimination of drugs, important factors to be considered when determining the appropriate dosage for pharmacological treatment. The ideal dose of most anticoagulants for obese people has not been established, including low molecular weight heparin and pentasaccharides. In this way, the therapeutic management of patients with acute coronary syndrome (ACS) and obesity is challenging and, consequently, can influence the efficacy and safety of anticoagulants.

The ideal dose of enoxaparin is calculated based on the patient's weight; however, as there is no distribution of the drug in fat, there is the possibility of excessive exposure to the anticoagulant effect in obese people, which contributes to the concern of supra-therapeutic concentrations when using a dose based on body weight.^4^ Fondaparinux is a synthetic pentasaccharide that acts as a selective inhibitor of factor Xa, with the advantage of a fixed dose, regardless of body weight. To date, few studies have been carried out in obese patients with the purpose of testing dosage adjustment, none of them in the ACS population.

In this edition of the *Arquivos Brasileiros de Cardiologia*, in a retrospective cohort study, Darzé et al. compared the use of enoxaparin and fondaparinux in 367 obese patients hospitalized with ACS, from 2010 to 2020. The primary outcome was defined as the combination of all-cause mortality, reinfarction, stroke, and major bleeding during hospitalization. The authors found no difference in the composite outcome, highlighting the possibility that fondaparinux could be as effective as enoxaparin without the need for dose adjustment.^5^

Some experts recommend measuring the anti-Xa factor in obese people, however, the minimum level of activity necessary for the treatment to be effective has not been well defined. Maximum anti-Xa target levels for twice-daily dosing are typically 0.5 or 0.6-1.0 IU/mL and can be used as a surrogate marker of therapeutic efficacy and safety.^4^ The first study to define an ideal dose was a case series described by Deal et al.^6^ who determined that the initial dose to achieve the ideal anti-Xa level would be 0.74 mg/kg in patients with an average BMI of 49.5 kg/m^2^. Subsequent studies defined optimal doses ranging from 0.70 to 0.81 mg/kg per dose.^7^ This variability is likely due to several factors: first, it appears that even within an obese patient population, those with higher BMIs require a lower total dose to achieve optimal anti-Xa levels, particularly in patients with BMIs greater than 50 kg/m^2^; secondly, the variability may result from the timing of the measurement of the anti-Xa^8^ level.

To answer the previous questions, Chilbert et al.,^9^ in a systematic review, evaluated anti-Xa levels in obese patients receiving enoxaparin therapy at the standard dose (≥ 0.95 mg/kg) versus reduced dosages that were grouped into <0.75 mg/kg (very low) and 0.75–0.85 mg/kg (low). Patients who received 0.75–0.85 mg/kg had better results in the therapeutic range with no apparent increase in thrombotic risk, while patients who received ≥0.95 mg/kg had an increase in bleeding events. If anti-factor Xa monitoring is indicated, the activity level should be determined three to five hours after the dose and only after the patient has received at least two doses. Strict clinical monitoring for signs and symptoms of complications, such as hemorrhage or thrombosis, is recommended, particularly in those weighing >150 kg or BMI >40 kg/m^2^.^10,11^

Given the scarcity of data, more studies are needed, such as the one carried out by Darzé et al.,^5^ to provide guidelines on the best approach for the appropriate use of anticoagulants in obese patients with ACS, thus ensuring optimization of clinical outcomes and patient safety. Enoxaparin offers the advantage of weight-adjusted dosing, which may be crucial in some cases but requires closer surveillance to avoid bleeding complications. Fondaparinux, with its fixed dosage and lower risk of bleeding, presents an attractive option, especially for patients at higher risk of bleeding events. Therefore, the therapeutic decision for the use of anticoagulants in obese patients must be supported by a holistic approach, considering the particularities with special attention to renal function, history of bleeding, and thrombotic risk, aiming to maximize therapeutic benefits and minimize risks.
